# Fabrication and properties of ZnO/GaN heterostructure nanocolumnar thin film on Si (111) substrate

**DOI:** 10.1186/1556-276X-8-112

**Published:** 2013-02-28

**Authors:** Xianqi Wei, Ranran Zhao, Minghui Shao, Xijin Xu, Jinzhao Huang

**Affiliations:** 1 School of Physics and Technology, University of Jinan, 250022, Jinan, People's Republic of China

**Keywords:** PLD, ZnO thin films, GaN buffer layer, Crystal structure, Optical properties

## Abstract

Zinc oxide thin films have been obtained on bare and GaN buffer layer decorated Si (111) substrates by pulsed laser deposition (PLD), respectively. GaN buffer layer was achieved by a two-step method. The structure, surface morphology, composition, and optical properties of these thin films were investigated by X-ray diffraction, field emission scanning electron microscopy, infrared absorption spectra, and photoluminiscence (PL) spectra, respectively. Scanning electron microscopy images indicate that the flower-like grains were presented on the surface of ZnO thin films grown on GaN/Si (111) substrate, while the ZnO thin films grown on Si (111) substrate show the morphology of inclination column. PL spectrum reveals that the ultraviolet emission efficiency of ZnO thin film on GaN buffer layer is high, and the defect emission of ZnO thin film derived from Zn_i_ and V_o_ is low. The results demonstrate that the existence of GaN buffer layer can greatly improve the ZnO thin film on the Si (111) substrate by PLD techniques.

## Background

ZnO is attractive for its various applications in electrical and optical devices by employing excitonic effects since it possesses promising wide and direct bandgap (3.37 eV at room temperature) and much larger exciton binding energy (60 meV) [1,2]. There has been considerable research interest in ZnO due to many potential applications in short wavelength for optoelectronic devices operating in the blue and ultraviolet (UV) region such as light-emitting diodes (LED) and gas-sensing applications [3]. It is found that a proper substrate is crucial to achieve a high-quality ZnO thin film and nanostructure [4]. Many substrates such as silicon, sapphire, quartz, etc. have been used to fabricate ZnO films [5-7]. Among these, Si is the most popular substrate due to its low cost, high crystalline perfection, and high productivity in large-area wafer. However, the large mismatch of lattice constants and thermal expansion coefficients between ZnO and Si will deteriorate the optical property of the ZnO films on Si substrates [8,9]. The employment of buffer layers such as GaN [10], MgO [11], and SiC [12] becomes a positive way to solve this problem. GaN is a perfect candidate because it has similar crystal structure as ZnO, and the lattice mismatch is 1.8 % on the *c*-plane; furthermore, the thermal expansion coefficients of ZnO are close to those of GaN. Recently, ZnO films have been grown on GaN template using molecular beam epitaxy, metal-organic chemical vapor deposition, magnetron sputtering, pulsed laser deposition (PLD), etc. [13-15]. Jang et al. [16,17] grew different ZnO nanostructures on GaN epitaxial layers via a hydrothermal method generating a variety of structures including rod-, sea urchin-, and flower-like structures. Studies on the growth of GaN-based and ZnMgO/MgO heterostructure materials have proved that column crystal growth is an effective way to relax part of the stress and improve the quality of the epitaxial layers [18-20]. That is, the formation of nanocolumnar microstructure allows the combination of materials with large lattice mismatch without generating dislocations, bringing on some novel low-dimensional physical phenomena.

In this paper, we obtained the novel ZnO/GaN heterostructure by growing GaN nanocolumns on Si substrate with PLD method, and then the annealing process was used to improve the crystallinity, respectively. The structure, surface morphology, composition, and optical properties of ZnO/GaN/Si thin films were investigated by X-ray diffraction (XRD), field emission scanning electron microscopy (FESEM), infrared (IR) absorption spectra, and photoluminescence (PL) spectra.

## Methods

### Samples and measurements

First, GaN thin films were grown on Si (111) substrate by PLD at the growth temperature of 800°C using a GaN ceramic target. The film deposition was carried out in a stainless steel vacuum chamber evacuated by a turbomolecular pump to a base pressure of 5.6 × 10^−5^ Pa. A pulsed Nd:YAG laser with a wavelength of 1,064 nm (repetition 10 Hz, duration 10 ns) was focused by a lens on the ZnO target at an angle of incidence of 45°. During the deposition, the laser incident energy was maintained at 300 mJ/pulse. The size of the ablation spot is about 0.5 mm in diameter. A series of Si (111) substrate was placed at 40 mm from the target surface. For the ZnO target ablation and even thin film fabrication, GaN target and substrate rotated reversely with a frequency of 7 rpm. GaN films were deposited in the nitrogen background of 1.3 Pa, and depositing time was 15 min. The thickness of GaN thin films measured is about 50 nm. Second, the samples were placed on a quartz carrier and annealed in a high-temperature tube quartz furnace. After the furnace reached the equilibrium temperature of 1,000°C the carrier with the GaN samples was placed in a constant temperature region of the furnace. Flowing N_2_ was introduced into the tube for 5 min at a flow rate of 100 ml/min to flush out the residual air. Then, we terminated N_2_ flow and introduced NH_3_ into the tube at a flow rate of 800 ml/min for 20 min. Finally, the NH_3_ was flushed out by N_2_ introduced into the tube for another 5 min before the carrier was removed from the furnace. Third, ZnO thin films were fabricated on GaN (111) template by PLD at a growth temperature of 400°C in O_2_ ambience with a pressure of 1.3 Pa using a ZnO ceramic target. The laser incident energy was maintained at 200 mJ/pulse, and depositing time was 60 min. The thickness of ZnO thin films is about 600 nm, which was measured by the weight technique. The structural properties of thin films were studied by Rigaku D/max-rB XRD (Tokyo, Japan) spectroscopy with Cu Kα line radiation at 0.15418 nm. The surface morphology and the microstructure were studied using FESEM (QUANTA 250, FEI Co., Hillsboro, OR, USA). The IR spectra were acquired using a BRUKER TENSOR27 spectrophotometer (Bruker Optik Gmbh, Ettlingen, Germany; wavenumber range 400 to 4,000 cm^−1^, optical resolution 4 cm^−1^, transmission mode). The optical properties of ZnO thin films were characterized by photoluminescence spectra with the excitation wavelength of 320 nm pumped by Xe lamp.

## Results and discussion

### Structural property of ZnO/GaN thin films

**Figure 1:**
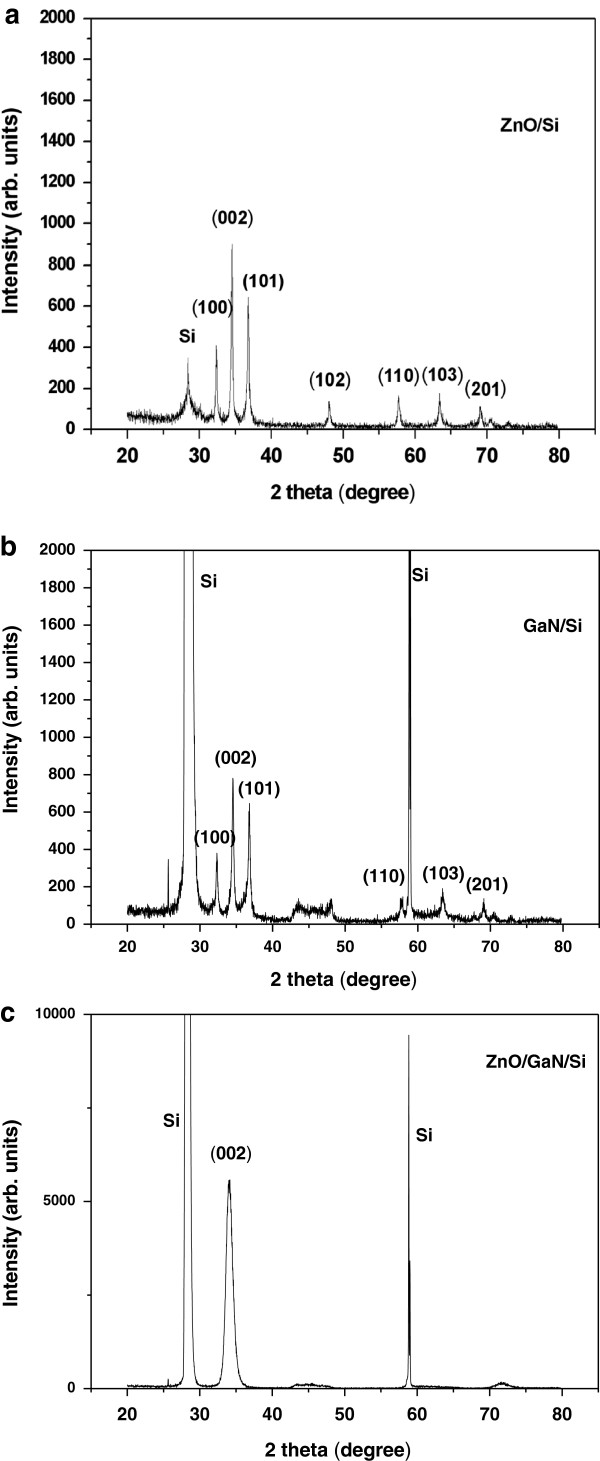
**XRD spectra.** ZnO films deposited on different substrates at 400°C: (**a**) Si substrate and (**c**) GaN/Si substrate. (**b**) Annealed GaN thin films deposited on Si substrate.

**Figure 2:**
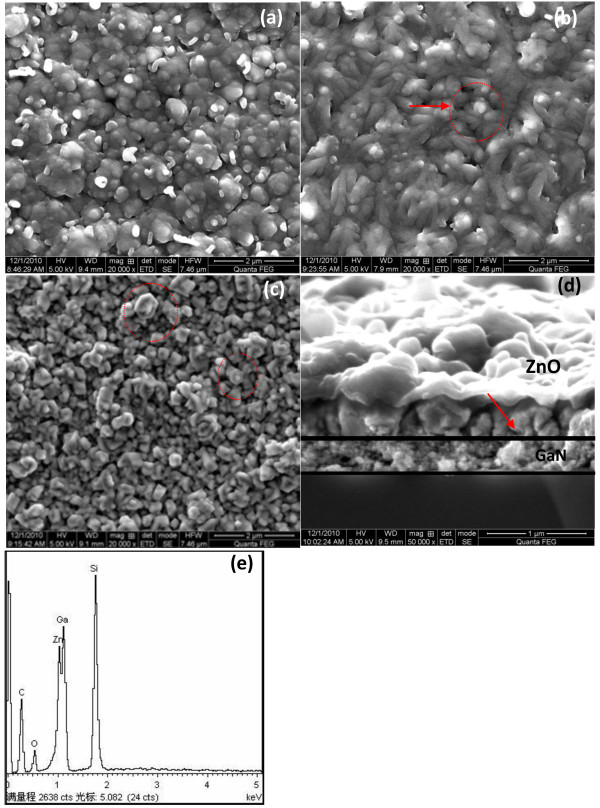
**SEM images.** ZnO films deposited on different substrates: (**a**) Si substrate and (**c**) GaN/Si substrate. (**b**) Annealed GaN thin films deposited on Si substrate at 800°C. (**d**) The cross-sectional images of the ZnO nanostructure on GaN/Si (111) substrates. (**e**) EDX spectrum of ZnO nanostructure derived from (**c**).

Figure 1a,b,c shows the XRD spectra of ZnO/Si, GaN/Si, and ZnO/GaN/Si films. Figure 1a shows that the reflection peaks of (100), (002), and (101) correspond to hexagonal ZnO with a wurtzite structure, but a preferred orientation along the (002) plane is intense. The diffraction peaks at 2*θ* = 34.55° owing to the dominant (002) GaN peak, 2*θ* = 32.39° owing to the GaN (100) peak, and 2*θ* = 36.86° owing to the GaN (101) peak could be observed in GaN/Si films as shown in Figure 1b. We noticed that the diffraction peak of (100) and (101) is significantly obvious as shown in Figure 1a,b. The reason is that the incline columnar grains are presented as shown in Figure 2a,b, and some ZnO and GaN nanostructures are not perpendicular to the substrate and partially exposed the (100) and (101) planes to the X-ray. Therefore, the diffraction intensity from the (100) and (101) planes is also rather strong in comparison with that of the other main planes, e.g., (110).

XRD peaks of ZnO films grown on GaN/Si substrate show merely (002) orientation, and an obvious promotion of crystalline quality of ZnO thin film grown on GaN/Si substrate can be obtained. Moreover, the (002) positions of ZnO and GaN show that the ZnO has very similar *c*-axis lattice parameter with GaN. The XRD pattern indicates that the growth direction of ZnO/GaN/Si is [002], and the orientation relationship with GaN epilayer is [002]_ZnO_//[002]_GaN_. This implies that ZnO (002) plane is synthesized parallel to the basal plane of the GaN epitaxial layer substrate.

### SEM observation

Figure 2a,b,c shows the SEM photographs of ZnO/Si films, GaN/Si films, and ZnO/GaN/Si films. Large and uneven grains are distributed on the ZnO surface for the thin film grown on Si (111) substrate as shown in Figure 2a. In Figure 2b, the incline columnar GaN structure annealed on the Si (111) substrate is presented. Besides, the obvious increase of crystalline grain with the hexagonal ZnO wurtzite structure is observed in Figure 2c; the incline columnar growth on the Si (111) substrate is transformed into a nanoflower grain on GaN/Si (111) template as shown in Figure 2c. Figure 2c illustrates that the surface property of ZnO/GaN/Si thin film is improved, and the thin film becomes more even than ZnO/Si film. It demonstrates that the quality of ZnO thin film was improved due to epitaxial growth of crystalline grain by GaN epitaxial layer. The reason is that the GaN buffer layer, especially nanocolumn, plays an important role for the epitaxial growth of ZnO thin films. As shown in Figure 2c, a lot of grains with hexagonal ZnO wurtzite structure can be observed. It is beneficial for growing high-quality epitaxial ZnO thin films on a GaN template. Figure 2d shows the cross-sectional images of the ZnO nanostructure on GaN/Si (111) substrates. The nanoflower ZnO nanostructure with the size of about 1 μm on the surface of thin film can be observed.

Compared with the growth of the most heterostructure with compact structure, the ZnO/GaN heterostructure interface in this study is loose, that is, the growth of ZnO nanostructure on GaN thin film with a column crystal. Also, the PL spectra of the ZnO grown on the GaN shows that the UV emission based on column crystal growth of ZnO has a higher emission efficiency and power than that grown with the conventional method.

From the EDX spectrum of ZnO nanostructure in Figure 2e derived from Figure 2c, the existence of the Zn and O peaks represented the elementary characterization of ZnO nanostructure. After the quasi-quantitative determination of the EDS spectrum, the weight percentages of O and Zn (K) were 38.23 and 11.98, respectively, and the atomic percentages of O and Zn (K) were 63.34 and 4.86, respectively. It is demonstrated that the purity of the fabrication is excellent without other residues (except C and Ga derived from the substrate and GaN buffer layer). It is also supposed that the ratio of Zn/O is more than 1 compared with that of the perfect chemical stoichiometry of ZnO. It reveals that there exists some O vacancy in the ZnO thin film.

### IR absorption spectra of ZnO thin film

**Figure 3:**
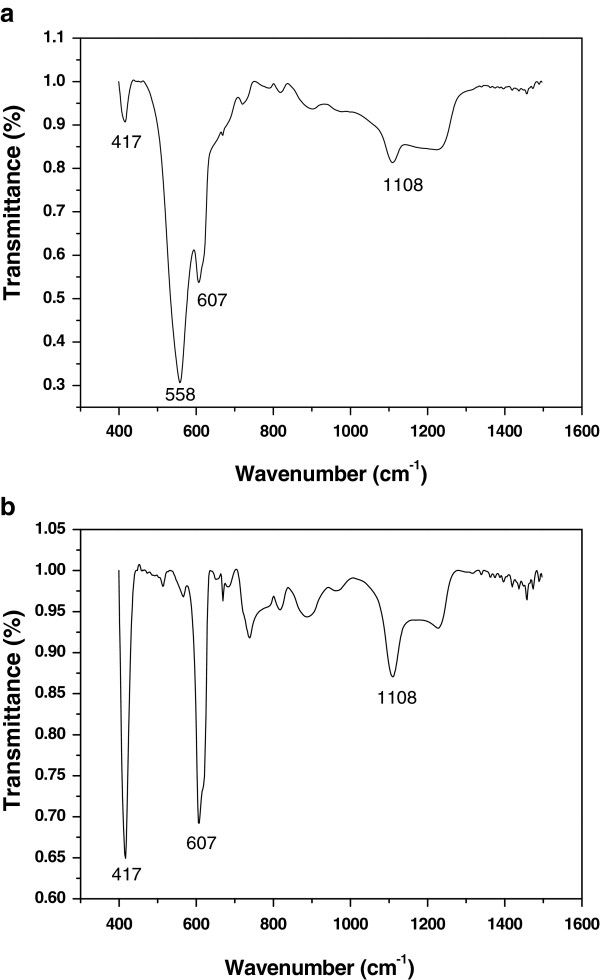
**IR absorption spectra.** (**a**) GaN/Si thin film and (**b**) ZnO thin film deposited on GaN/Si substrate.

The IR absorption spectra of GaN/Si and ZnO/GaN/Si films deposited at a deposition temperature of 400°C are given in Figure 3a,b, respectively. An intense and broad band at 558 cm^−1^ corresponding to the stretching vibration absorption of Ga-N bonds in a hexagonal GaN crystal can be observed as shown in Figure 3a [21]. The absorption band at a wavenumber of 607 cm^−1^ is a local vibration of substitutional carbon in a Si crystal lattice [22,23]. A weak peak sited at 1,108 cm^−1^ is a vibration absorption of Si-O bond [24]. The weak absorption peak sited at 414 cm^−1^ may be derived from the vibration absorption of Ga-O bond formed when GaN thin film was annealed or cooled down. In Figure 3b, the spectrum contains three absorption bands at wavenumbers 417, 558, and 607 cm^−1^, respectively. The band located at 417 cm^−1^ is a typical ZnO absorption attributed to the bending vibration absorption of Zn-O bond, which corresponds to the E_1_ symmetry transverse optical phonon mode, and the absorption intensity is increased obviously. The reason should be the ZnO thin film fabricated on GaN/Si substrate with perfect nanostructure, while the film deposited on Si substrate presents merely the *c*-axis orientation growth. The observation of IR absorption spectra shows that the ZnO thin film fabricated on GaN substrate improves the crystalline quality.

### Photoluminescence spectra

**Figure 4:**
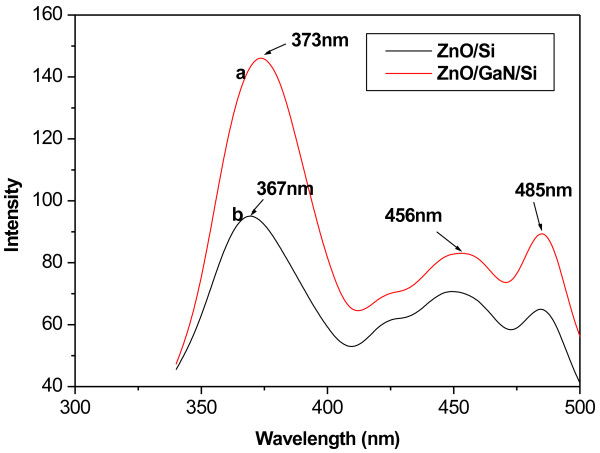
**PL spectra of ZnO thin film deposited on different substrates at 400°C.** (**a**) Si substrate and (**b**) GaN/Si substrate.

Figure 4 (a) shows the PL spectrum of ZnO films fabricated at 400°C using GaN buffer layer, and Figure 4 (b) shows the PL spectra of ZnO/Si thin film grown at 400°C. Figure 4 shows three main emission peaks. One intense peak centered at 373 nm is near-band emission, which corresponds to the exciton emission from near conduction band to valence band. Another weak one located at 456 nm is defect emission. As shown in Figure 4, merely the weak defect emission band centered at 456 and 485 nm can be observed in two thin films. This blue emission located at 456 nm most likely derives from electronic transition from the donor level of Zn interstitial to acceptor energy level of Zn vacancy according to Sun's calculation by full-potential linear muffin-tin orbital method [25-27]. This shows that some Zn_i_ atoms exist in fabricated ZnO thin films. The emission located at 485 nm may be caused by the electronic transition between the anti-oxygen (O_Zn_) and the conduction band. The PL spectra in Figure 4 (a) show that the UV emission of ZnO thin film fabricated on GaN/Si substrate is higher than that fabricated on the Si substrate. The ratio of intensity of UV emission of ZnO/GaN/Si film to that of ZnO/Si film is about 2:1, and the ratio of FWHM of UV peak of ZnO/GaN/Si film to that of ZnO/Si film is about 7:11.

As shown in Figure 4 (a), the UV emission located at 367 nm is increased, and the visible emission at 456 nm is decreased. The increase of UV emission and the decrease of the defect emission indicate that the structure of ZnO/GaN/Si thin film becomes more perfect. The UV peak appears as a redshift from 367 to 373 nm. The relaxation of interface strain is the main reason because of the formation of ZnO/GaN/Si heterostructure. The PL spectra of ZnO thin film fabricated on two different substrates show that the PL property of thin film fabricated using GaN buffer layer is more superior to that of ZnO/Si film. The ratio of visible emission of ZnO thin film fabricated on Si substrate is high, indicating that more defects exists in ZnO thin film. This is consistent with the analysis of two XRD spectra of ZnO thin films above.

## Conclusion

ZnO thin films have been fabricated on GaN/Si and Si (111) substrates at the deposited temperature of 400°C, respectively. The structural and optical properties of ZnO thin films fabricated on different substrates are investigated systematically by XRD, FESEM, FTIR, and PL spectra. The FESEM results show that the ZnO/GaN/Si film is two-dimensionally grown with flower-like structure, while the ZnO/Si film is the (002) orientation grown with an incline columnar structure. The GaN buffer layer plays an important role for the transformation of the growth mode of ZnO thin films from one-dimensional to two-dimensional. The high UV emission of ZnO/GaN/Si films and the less defect emission with Zn_i_ and V_o_ reveal that the film possesses high quality by introducing a GaN buffer layer on the Si (111) substrate. In conclusion, it is favorable to fabricate high emission efficiency ZnO thin film on GaN/Si substrate rather than Si (111) substrate. The study provides an opportunity for constructing the nanopillar array ZnO/GaN heterostructure and deep UV emission LED devices.

## Competing interests

The authors declare that they have no competing interests.

## Authors' contributions

XW and XX proposed the research work, coordinated the collaboration, carried out the analyses of experimental results, and drafted the manuscript. JH designed the experiment and experimental setup and carried out the measurements. RZ and MS participated in experimental measurements, results and discussion, and analyses. All authors read and approved the final manuscript.
